# Decision-Making in Health and Fitness

**DOI:** 10.3389/fpubh.2019.00006

**Published:** 2019-01-23

**Authors:** Philip B. Maffetone, Paul B. Laursen

**Affiliations:** ^1^Independent Researcher, Ormond Beach, FL, United States; ^2^Sports Performance Research Institute New Zealand, Auckland University of Technology, Auckland, New Zealand

**Keywords:** chronic disease, consumer choice behavior, emotion reactivity, system 1 and system 2, health education, diet, exercise

## Introduction

Lifestyle choices associated with food and exercise habits are fundamentally a complex decision-making process associated with many biological, social, and emotional variables. As this may be considered more difficult and time consuming, many people choose to make the simple straightforward and emotional decision influenced primarily by marketers and social media, giving consumers the perception of quick, positive predictable outcomes, even if they are inaccurate and appear too good to be true. Rather than a lack of consensus by scientists and clinicians on how to improve health and fitness, poor choices by consumers encouraged by advertisements and social trends may contribute to the continued growth of chronic illness and disability that leads to higher healthcare costs. Within this framework, modern decision-making theory may help us better understand this global problem.

Marketers selling health and fitness products and services have long since seized on our tendency to respond to advertisements that promise quick-fix solutions—especially diet and exercise fads that speak to the emotionally-run limbic system and easily grab consumer attention. Unfortunately, these initiatives often prevent people from thinking about the potential benefits and risks of using such products and services, which requires a more complex decision-making cognitive process to make the same choice. *Weight loss, injury prevention*, and *increased energy* are among the common buzzwords that quickly receive consumer's attention. Terms like *fresh, natural*, and *local*, which don't necessarily imply healthy, along with many certified organic food items, can in fact be classified as junk food. These quick-fix choices often result in postponing improved health and fitness for an individual, with wide-ranging negative outcomes; consider the current overfat pandemic with its downstream diseases and disabilities in the US, where, despite rising exercise rates, 91% of adults are now affected (1, 2).

Since food and exercise are known to significantly influence health and fitness, and impact the development of chronic disease, disability, and premature death (3), the processes by which individuals make lifestyle choices—and their related consequences—should be an important public health concern.

## Cognitive Decision-Making

Denes-Raj and Epstein (4) suggest that decision-making behavior is guided by two different cognitive processes, the first being an emotional response typical of interpersonal interactions, and the second an analytical response such as that used to solve a mathematical problem. The theory was simplified further by Amos Tversky, with Stanovich and West naming the emotional process “System 1” and the rational one “System 2” (5, 6). Kahneman applied these ideas to *economic* behavior (7), with Tversky and Kahneman awarded separate Nobel prizes for their respective works. The application of System 1 and System 2 decision-making behavior in the context of health and fitness can have wide-ranging potential personal and global public health implications, and is described here as *behavioral health and fitness*. [*Health* is defined as all areas of the body working in harmony, while *fitness* is the ability to perform physical activity (8)].

Large numbers of people around the world attempt to regularly manage a variety of personal health and fitness routines. At its onset, this self-care process can be strongly influenced by companies selling products and services (diets, books, programs, exercise equipment) through radio and TV, online and print media, health, and fitness societies/agencies and from governmental recommendations, the latter two strongly influenced by politics and lobbying. The process is often void of individuality, encourages a one-size-fits-all notion, and can lead to dangerous herd behavior (9). These are associated with a System 1 response. Personalizing food and exercise choices require more thinking and is associated with System 2.

## Characteristics of System 1 and System 2

Normally, both modes of decision-making are used in our day-to-day lives, and both have potential value. Consider System 1's first impression, an often accurate assessment of another person, place, food and physical activity. This impression may correspond to one's System 2 analysis over time. However, more often the use of images, words, sounds and other impressions in marketing, quickly sway people by enlisting System 1 to help sell unhealthy products and services.

### System 1

Involving simple everyday choices that are habit- and reaction-based, usually made with little thinking, attention, or information, System 1 governs the quick decisions such as which of several doors to use when entering an office building, lanes to take on a highway, or seats to sit in at an airport. However, important decisions that can impact on immediate and long-term individual and population health and fitness are influenced if not governed by System 1 as well (10).

The System 1 process is primarily an unconscious but natural reaction, such that one's true underlying attitude or motivation for the decision is hard to come by, and the individual will likely provide one of several plausible rationalizations to justify how they made the decision. While this system is leveraged particularly well by marketers advertising products and services, it comes with the potential for strong bias and error referred to as cognitive illusions that can lead to reduced health and fitness. Fleeting first impressions appear attractive to System 1 and predominate its decision-making: Seeing a splashy colorful cover of a new diet book or a smiling lean person working out are common examples.

### System 2

Relying on conscious intellect for lifestyle decision-making, System 2 requires more time to assess a particular eating plan or exercise program. In terms of self-care, it also provides an individual with the ability for ongoing monitoring of signs and symptoms that measure progress.

The more reliable and logical System 2 process can yield a personalized approach rather than a one-size-fits-all menu, and grants the ability to incorporate a planned, flexible program that can lead to improved outcomes (11). Requiring reasonable literacy, this approach offers greater autonomy, and can also reduce healthcare costs (12).

**Figure 1:**
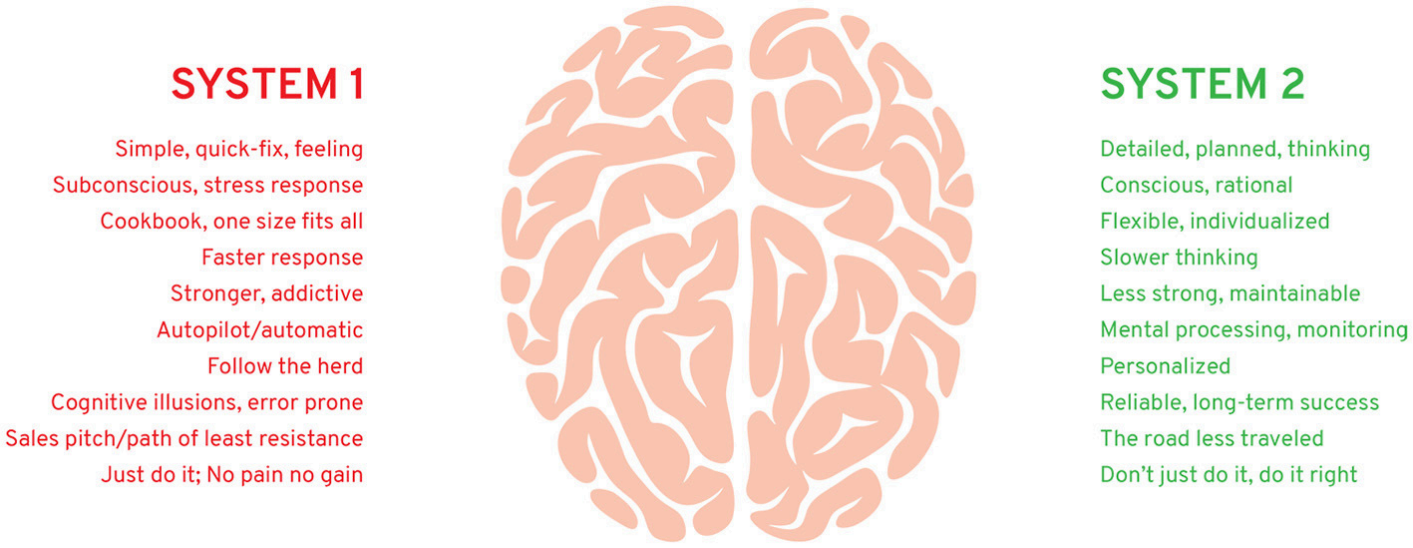
Some factors associated with behavioral health and fitness.

Figure 1 lists some factors associated with System 1 and System 2 decision-making.

Health practitioners can also play an important part in teaching patients about the lifestyle habits associated with their particular needs, helping them avoid making irrational or poor choices (3, 13). However, for the benefits of health education to succeed, a high level of engagement is required. Here again, this may be impaired by society's System 1 dominance in the health and fitness arena, where consumers—patients and practitioners alike—are influenced. Unfortunately, few practitioners provide details on decision-making and modification of behavior for other related reasons: it's time-consuming, most practitioners are not knowledgeable enough, and patients are given few strategies for maintenance. Likewise, governmental recommendations are extremely simplistic, not individualized, and without encouragement.

## Costs of System 1

System 1 marketing deception has been a successful business strategy for decades, selling untold numbers of health and fitness products and services that promise quick improvement that System 2 thinks is unlikely. For example, the diet industry in Europe and the United States alone has annual revenues in excess of $150 billion, and rising, yet up to two-thirds of any weight lost is regained within 1 year—and almost all is regained within 5 years, along with lost health (14).

Downstream healthcare costs continue to be high and are rising globally as well. In the US, 2014 health-care costs climbed to $3.2 trillion (15), with the Kaiser Family Foundation estimating a worldwide cumulative healthcare loss of $47 trillion between 2011 and 2030.

## Examples and Misconceptions

Here, we provide two examples of how the reliance of a System 1 approach can lead to failure:

1. A person wanting to lose weight is attracted to a program claiming you can shed 10 pounds the first week. Whether initially successful or not, the diet usually fails to provide long-term results, and may cause side effects such as nutritional imbalance, metabolic impairment, and disordered eating.
2. A person wants to exercise to get into shape. Regular gym workouts encouraged by the no pain, no gain philosophy pushes the process. After a period of initial excitement, with some results realized—lost weight, more fitness—fatigue, soreness, injury, and frustration may develop causing some to give up working out. Others may become addicted to exercise, and despite pain or frustration, continue pushing through it, increasing stress hormones that impair health and fitness.

System 1-based marketing has spawned many popular misconceptions, trendy fads, and rally cries that become unhealthy social mantras. Below are two popular and very successful examples:

1. *No pain, no gain*. Perhaps the first social description of no-pain no-gain came from Benjamin Franklin in his writings on capitalism (16). But in the fitness arena, this rallying cry glorifies pain and the high rates of preventable injury. It overshadows the scientific consensus (System 2), considered more effective and healthy. Bill Bowerman, legendary sports coach and co-founder of Nike, said, “The idea that the harder you work, the better you're going to be is just garbage. The greatest improvement is made by the man or woman who works most intelligently.”
2. *Just do it*. Ironically, this popular Nike ad slogan, which appeared later in the company's evolution, communicates the System 1 message that it is enough to simply make a snap judgment to follow a certain exercise ritual without further consideration, encouraging a herd mentality (9). System 2 might think, *don't just do it, do it right*.

## The New Players

Mobile trackers are the relatively new players in the health and fitness arena, and enlist primarily System 1 due to their emphasis on gaming and gamification. As they collect largely irrelevant data, users tend to give up on them within 6 months (17). Despite this, analysts at Morgan Stanley believe these devices will become a $1.6 trillion business in the near future (18). Indeed, the System 1 slant of mobile trackers, in the absence of more substantive and sophisticated analytics that engage System 2 thinking, may contribute to their early abandonment and demise: there is little reason to continue engaging the user through System 2 once System 1 thinking has run its course, at which point the user moves on to the next new device or program that captures the attention of System 1.

## A Public Health Choice

The purpose of public health includes informing and educating the public, mobilizing community partnerships, developing policies to support health goals, and enforcing related laws and regulations (19). Despite the reality that many consumers use System 1 thinking to make unhealthy lifestyle choices, public health officials, health practitioners, policy makers, and others must work out how best to interact with an existing System 1 process to reverse this trend (13, 20). Exploiting System 1 can help make health and fitness habitual, a process accomplished many times with whole populations reducing health-related risks through public health actions. Wide et al. (21) showed that a brief psychological intervention in young adults with a high prevalence of dental caries led to an immediate positive effect with improved oral health behaviors. The use of seatbelts has significantly reduced injury and death in vehicular accidents due to laws, high visibility enforcement, and fines, and promoting positive beliefs (22). The importance of hand washing education to help prevent infections has occurred throughout most populations (23). Promotion of self-care has also been effective in such areas as breast cancer screening behavior (24), and gestational anemia (25). While we applaud these and other public health successes, improved behavioral health and fitness promotions are urgently needed, while reducing the advertisement of unhealthy products and services to avoid drowning out the positive recommendations.

## Recommendations

More specific suggestions to encourage individuals to avoid making poor diet and exercise choices can be made through two general public health approaches. First is to further restrict or ban the advertising and promotion of unhealthy products and services. This is being achieved with tobacco, and is gradually being implemented now by a ban on soda sales in some schools or junk food in some hospitals, and/or through a higher tax on unhealthy products. Second, and concurrent, is the promotion of healthy options, which can also include reductions or elimination of tax on healthy foods such as fruits and vegetables. These can be attempted through System 2 approaches but simplified sufficiently for most people to understand, implement, and maintain. This strategy may also require more creative, simple System 1-type guidelines, not unlike traditional successful marketing, to encourage easier understanding and behavioral changes. In addition:

- Public health communication messages and campaigns should be more clear and modernized; the Institute of Medicine found a major mismatch between the health information people receive and what they understand (26).
- These lifestyle recommendations should also be updated regularly as they can quickly become outdated (27). For example, the US government has just updated recommendations for physical activity for the first time in 10 years; compared with this once a decade frequency, companies promoting unhealthy products and services are bombarding consumers on a daily basis (28).
- The promotion of education strategies has already been successfully applied to individuals performing self-care for such conditions as cardiovascular disease (29) and mild cognitive impairment (30). With a sufficient level of scientific consensus in the area of diet and exercise, similar strategies regularly implemented can help people make better choices and offset ongoing System 1 misinformation campaigns.

There is no doubt that lifestyle change is difficult, one created in great part by decades of harmful System 1 marketing. This also can feed poor self-discipline in consumers. However, with the added awareness of behavioral health and fitness, combined with the help of public health actions, the process of self-care that many consumers follow could improve discipline and intellectual judgment as part of a System 2 process that more likely brings long-term success.

When it comes to making lifestyle choices, large numbers of people around the world who practice self-care are guided by System 1 thinking primarily from corporate marketing of health and fitness products and services that have potentially grave, unhealthy consequences. This may be significantly influencing the corresponding rise of chronic disease, physical impairment, lowered mental health, reduced quality of life, and healthcare costs. It is our hope that this article could help further increase public health awareness and stimulate a more detailed plan of action for effective strategies to improve and maintain health and fitness behavior, and consequently reduce mortality and morbidity of chronic disease and disability in adults and children.

## Author Contributions

PM conceived the idea for the manuscript and lead the authorship process. PL edited a draft of the manuscript and contributed to the content.

### Conflict of Interest Statement

PM is an independent clinical consultant, writes articles, and books that include the topics presented herein, and has a business website pertaining to health and fitness (www.philmaffetone.com). PL is an independent consultant, writes articles and books, and has a website pertaining to performance, health, and longevity (www.plewsandprof.com).
